# Supplementary material for: Characterization of Regulatory and Transporter Genes in the Biosynthesis of Anti-Tuberculosis Ilamycins and Production in a Heterologous Host
Source: Mar Drugs. 2020 Apr 17;18(4):216. doi: 10.3390/md18040216 (PMC7230496; doi:10.3390/md18040216)
Supplement: Supplementary file 1 [file marinedrugs-18-00216-s001.pdf]

Supplementary Materials

Characterization of Regulatory and Transporter Genes in the Biosynthesis of Anti-Tuberculosis Ilamycins and Production in a Heterologous Host

Jianqiao He [1,3], Xin Wei [1,3], Zhijie Yang [1,3], Yan, Li, [1] Jianhua Ju [1,2,3] and Junying Ma [1,2*]

[1] CAS Key Laboratory of Tropical Marine Bio-resources and Ecology, Guangdong Key Laboratory of Marine Materia Medica, RNAM Center for Marine Microbiology, South China Sea Institute of Oceanology, Chinese Academy of Sciences, Guangzhou, 510301, China; weixin1011@126.com (X.W.); 13409727100@163.com (J.H.); yzjie6@126.com (Z.Y.)

[2] Key Special Project for Introduced Talents Team of Southern Marine Science and Engineering Guangdong Laboratory (Guangzhou).

[3] University of Chinese Academy of Sciences, Beijing, 100049, China.

* Correspondence: majunying@scsio.ac.cn; Tel.: (+86)20-34066449 (J.M.)

Received: 20 March 2020; Accepted: 14 April 2020; Published: 17 April 2020

Item Name	Contents/Description	Page
**Table S1**	Summary of strains and plasmids used in this study.	S2
**Table S2**	Summary of primers used in this study.	S3
**Figure S1**	Disruption of *ilaA* in *S. atratus* SCSIO ZH16.	S4
**Figure S2**	Disruption of *ilaB* in *S. atratus* SCSIO ZH16.	S4
**Figure S3**	Sequence alignment of IlaJ with its homologues.	S5
**Figure S4**	Secondary structure prediction of transmembrane region of IlaK with TMHMM V2.0 and primary sequence alignment of IlaK with its homologues from the genus *Streptomyces*.	S6
**Figure S5**	Disruption of *ilaJ* in *S. atratus* SCSIO ZH16.	S7
**Figure S6**	Disruption of *ilak* in *S. atratus* SCSIO ZH16.	S7
**Figure S7**	Disruption of *ilaJk* in *S. atratus* SCSIO ZH16.	S8
**Figure S8**	The quantitative HPLC standard curve for ilamycin D.	S8
**Figure S9**	The PCR verification of PAC-7A6 containing entire *ila* gene cluster.	S9
**Reference**		S9

Table 1. Summary of strains and plasmids used in this study.

**Strains/ plasmids**	**Relevant phenotype**	**Source/ [Ref]**
S. atratus SCSIO ZH16	Wild-type (WT) producer of ilamycin	[1]
S. coelicolor M1152		[2]
Δ*ilaA*	*S. atratus* SCSIO ZH16 with a 296 bp of *ilaA* substituted by *aac(3)IV*+OriT	This work
Δ*ilaB*	*S. atratus* SCSIO ZH16 with a 816 bp of *ilaB* substituted by *aac(3)IV*+OriT	This work
Δ*ilaJ*	*S. atratus* SCSIO ZH16 with a 723 bp of *ilaJ* substituted by *aac(3)IV*+OriT	This work
Δ*ilaK*	*S. atratus*SCSIO ZH16 with a 471 bp of *ilaK* substituted by *aac(3)IV*+OriT	This work
Δ*ilaJK*	*S. atratus*SCSIO ZH16 with a 1502 bp of *ilaJ and ilaK* substituted by *aac(3)IV*+OriT	
E. coli		
Bw25113	K-12 derivative: *araBAD, rhaBAD*	[3]
ET12567	*dam, dcm, hsdM, hsdS, hsdR, catR , tetR*	[4]
Plasmids		
pIJ773	P1-FRT-oriT-aac(3)IV-FRT-P2	[5]
pIJ790	λ-RED (*gam bet exo*) CmlR *araCrep101ts*	[5]
pUZ8002	*tra*, *neo*, RP4	[6]
pL646ATE	*Tsr, acc(3)IV, ermE*P*	[7]
cosmid-23D	A cosmid which contains partial ilamycin biosynthetic gene cluster	This work
cosmid-42G	A cosmid which contains partial ilamycin biosynthetic gene cluster	This work
PAC-7A6	A cosmid which contains complete ilamycin biosynthetic gene cluster	This work
*p*Δ*ilaA*	A 296 bp fragment in *ilaA* in cosmid 23D was substituted by the *aac(IV)*+OriT cassette using the PCR-targeting strategy	This work
*p*Δ*ilaB*	A 816 bp fragment in *ilaB* in cosmid 23D was substituted by the *aac(IV)*+OriT cassette using the PCR-targeting strategy	This work
*p*Δ*ilaJ*	A 723 bp fragment in *ilaJ* in cosmid 42G was substituted by the aac(IV)+OriT cassette using the PCR-targeting strategy	This work
*p*Δ*ilaK*	A 471 bp fragment in *ilaK* in cosmid 42G was substituted by the *aac(IV)*+OriT cassette using the PCR-targeting strategy	This work
*p*Δ*ilaJK*	A 1502 bp fragment in *ilaJ* and *ilaK* substituted by the *aac(IV)*+OriT cassette using the PCR-targeting strategy	This work
ΔilaB::ilaB	An integrated vector pL646ATE with complete *ilaB* for complementation of Δ*ilaB* mutant	This work
S. atrratus ZH16:*ilaB*	An integrated vector pL646ATE with complete *ilaB* for over-expression	This work

Table S2 the primers were used for gene inactivation and verification.

Primer name	The sequence (5'-3')	purpose
Del*ilaA*F	GTGCTGACGAATGCCCTGTACAGGATCTTCATCTTG ACAATTCCGGGGATCCGTCGACC	For disrupting *ilaA*
Del*ilaA*R	TCACCGCGCCGAACCGGCGTAAACGGAATCAGGC GCCGCTGTAGGCTGGAGCTGCTTC	
Del*ilaB*F	GACGTGCCGGGACGGGGCAGCGGGACGGGTACGT CCTCGATTCCGGGGATCCGTCGACC	For disrupting *ilaB*
Del*ilaB*R	GAGACGGGCCACGACCTCCTGACGGTGAGGCGGG ACCGCTGTAGGCTGGAGCTGCTTC	
Del*ilaJ*F	GCCGTCGACGGCCTCGATCTGGCCGTCCCGGCCGG TGCCATTCCGGGGATCCGTCGACC	For disrupting *ilaJ*
Del*ilaJ*R	GACGGTCTGGGTGAGTTCGCCCAGCGCACGCGCCA CGCTTGTAGGCTGGAGCTGCTTC	
Del*ilaK*F	GTCGAGGCGGACGAGAGGCTGCGCCGGGTCCTGG GTGAGATTCCGGGGATCCGTCGACC	For disrupting *ilaK*
Del*ilaK*R	GCGCAGCACCAGGCCGAGGAACGTCCAGATCCAC GAGACTGTAGGCTGGAGCTGCTTC	
ID*ilaA*F	AGGGTCATCATCGCTGTCTCG	For verifying mutant of Δ*ilaA*
ID*ilaA*R	CGGCATGGGCTTTCAATCTAC	
ID*ilaB*F	CGACGGGGTCACAACATCCT	For verifying mutant of Δ*ilaB*
ID*ilaB*R	CATTTCCTCCGACGCACGATC	
ID*ilaJ*F	GCGAACCTAAGGGTGAATGTG	For verifying mutant of Δ*ilaJ*
ID*ilaJ*R	GGTCAGGGCGAGGAACAC	
ID*ilaK*F	ACGACGACCGAGGAGACCC	For verifying mutant of Δ*ilaK*
ID*ilaK*R	CGAATGCCTTCAGCCACCC	
Com-ilaB-F	aaaa*catatg*ATGATCGGTAGATTGAAAGCCCATGC	For complete cloning the *ilaB*
Com-ilaB-R	aaaaa*ctagtggatcc*TCACCCCGCCTCCGTCG	
orf(-2) F	CGGTGCGTGATGAGATCCTGT	For verification of the heterologues expression conjugants
*orf(-2)*R	TCGAACTTCCCGAGCAAACG	
*ilaN*F	CCGCTCGCCGTCTTCATCG	For verification of the heterologues expression conjugants
*ilaN*R	TGAGTCGTCGCCCGCCTTC	
*orf(+2)*F	ACAGAGCGGATTCCGTGGTG	For verification of the heterologues expression conjugants
*orf(+2)*R	AGCGATCTTGTGGGTTCAGG	

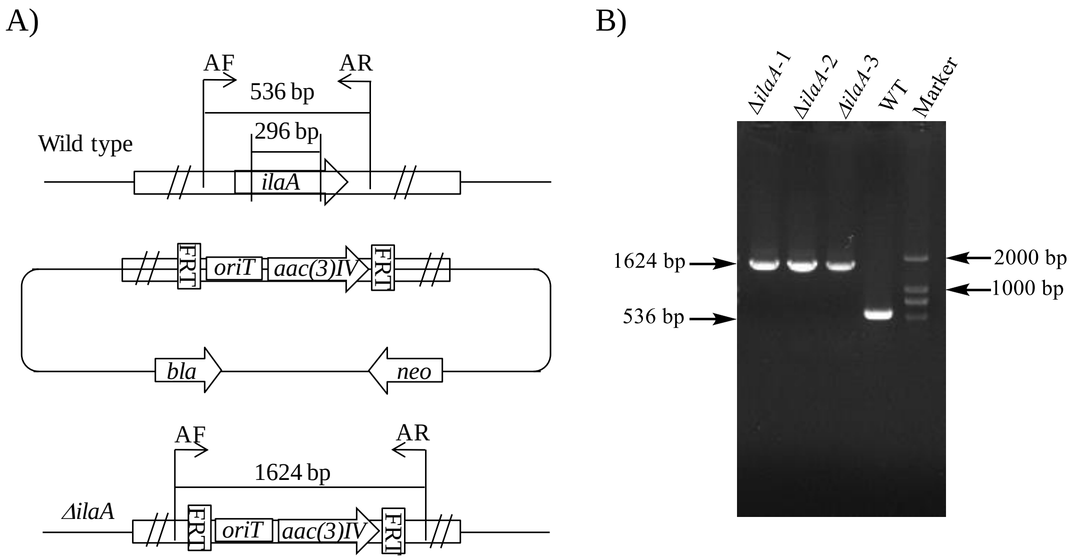

Figure S1. Disruption of *ilaA* in wild type *S. atratus* SCSIO ZH16 via PCR-targeting. (A) Schematic representation for disruption of *ilaA*. (B) PCR analyses of the WT strain and the *ilaA* double-cross mutant carried out using the primers listed in Table S2. M: DNA molecular ladder; WT: using the genomic DNA of *S. atratus* SCSIO ZH16 as template; Δ*ilaA*-1-3: using the genomic DNA of *ilaA* mutant as template.

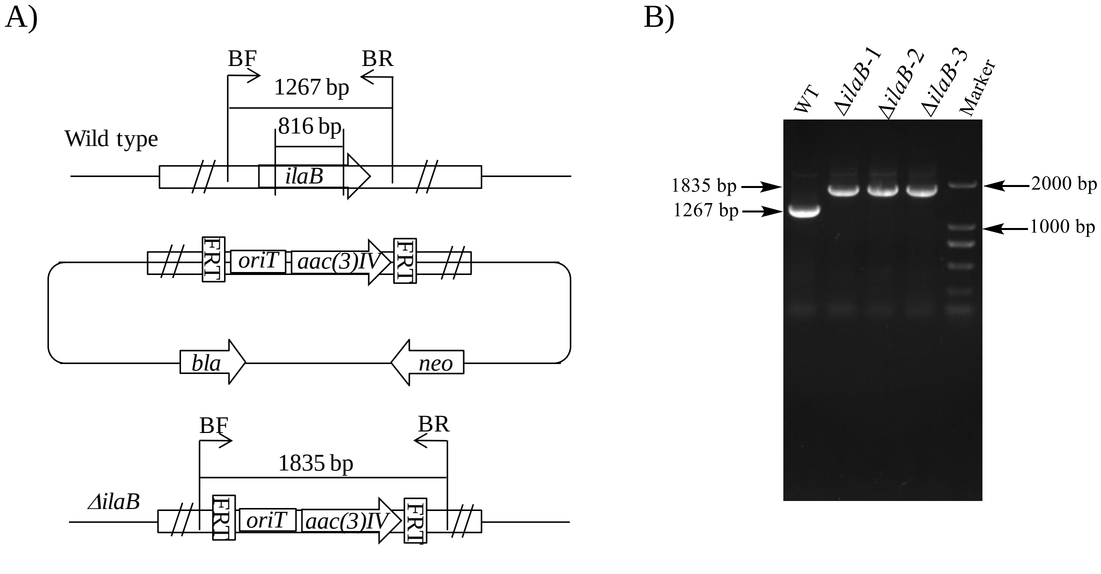

Figure S2. Disruption of *ilaB* in wild type *S. atratus* SCSIO ZH16 via PCR-targeting. (A) Schematic representation for disruption of *ilaB.* (B) PCR analyses of the WT strain and the *ilaB* double-cross mutant carried out using the primers listed in Table S2. M: DNA molecular ladder; WT: using the genomic DNA of *S. atratus* SCSIO ZH16 as template; Δ*ilaB*-1-3: using the genomic DNA of *ilaB* mutant as template.

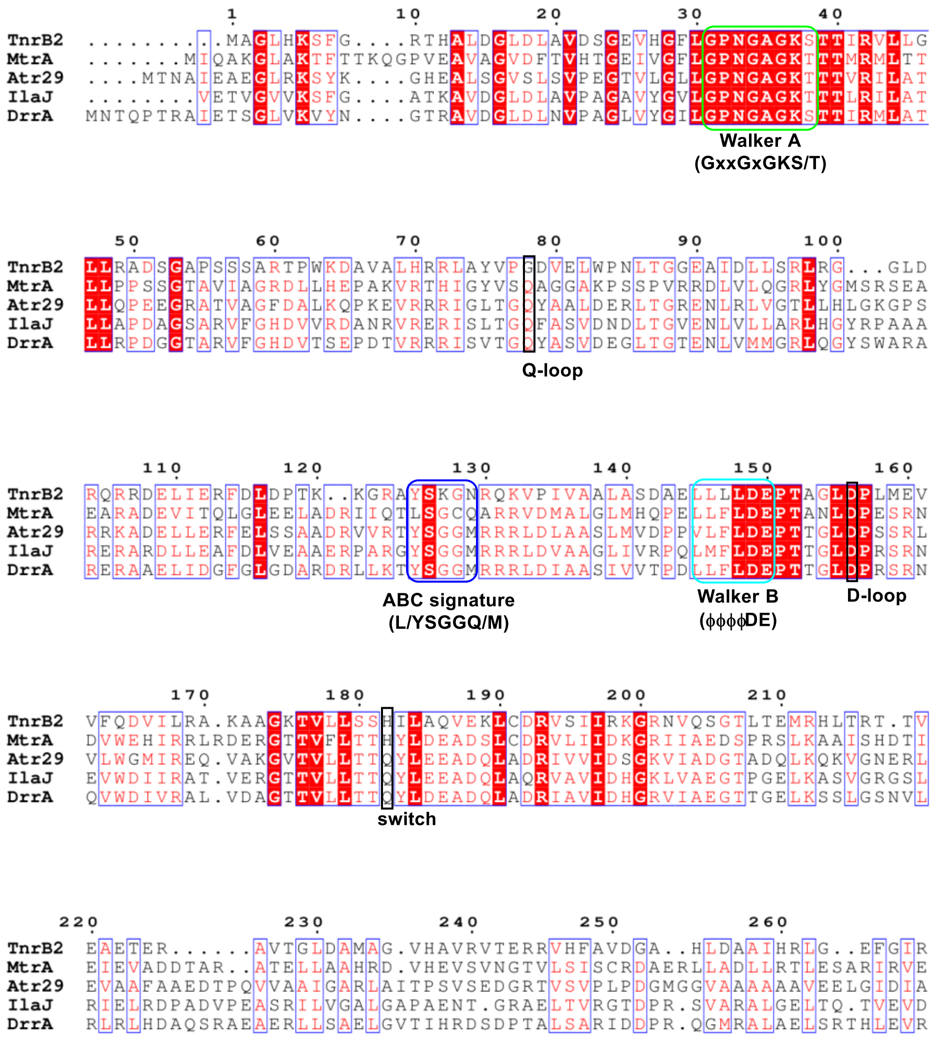

Figure S3. The primary sequence alignment of IlaJ its homologues from other strains including MtrA (GenBank accession no. CAK50797) from *Streptomyces argillaceus*, Atr29 (GenBank accession no. QBG38790.1) from *S. atratus* SCSIO ZH16, DrrA (GenBank accession no. ATW50556.1) from *Streptomyces peucetius subsp. caesius* ATCC 27952. The predicated Walker A, Walker B, the ABC signature motif, the Q-loop, the D-loop and the switch region were marked with fluorescent green box, cyan box, blue box and black box.

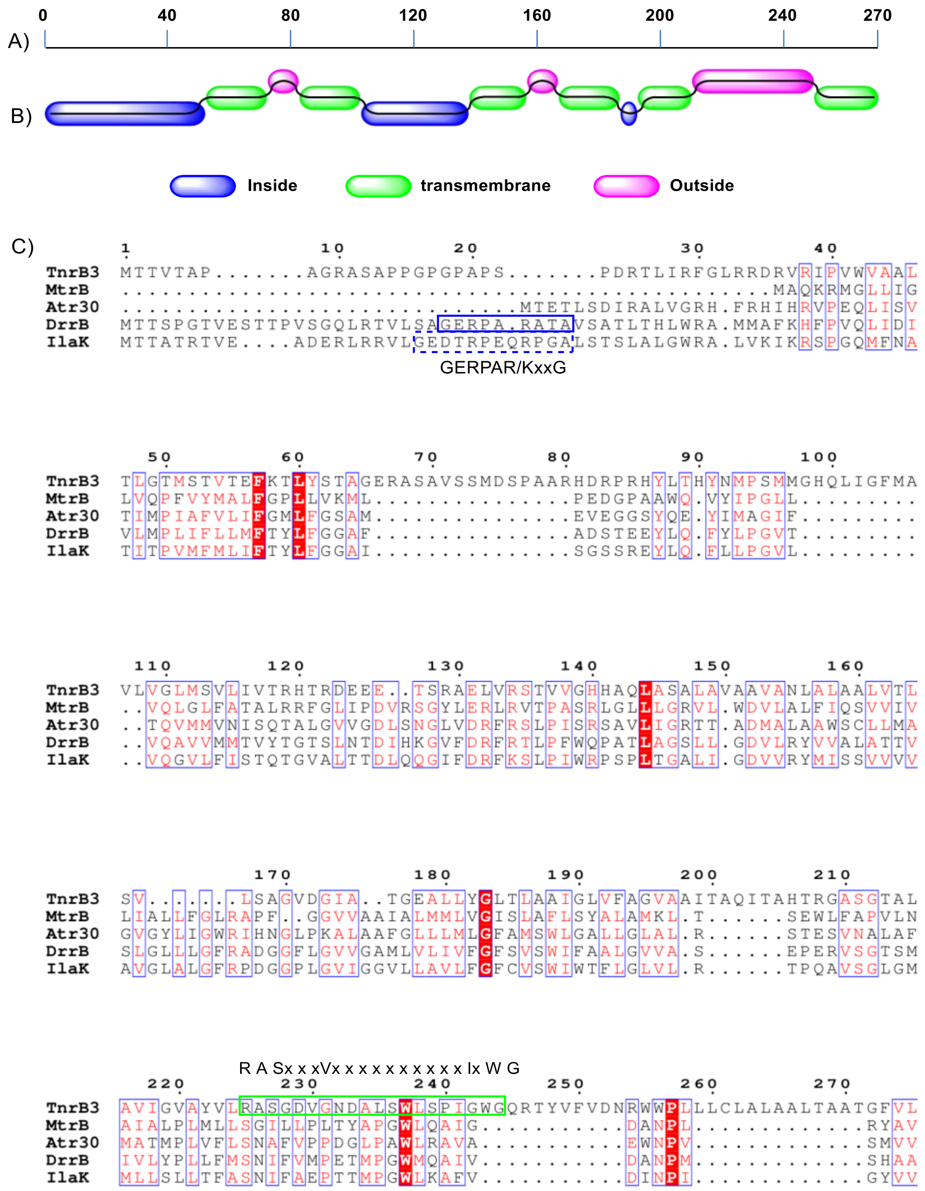

Figure S4. Secondary structure prediction of transmembrane region of IlaK with TMHMM V2.0 and primary sequence alignment of IlaK with its homologues from the genus *Streptomyces*. A) the protein length of IlaK; B) the transmembrane region of IlaK; C) the sequence alignment of IlaK with its homologues including TnrB3 (GenBank accession no. CAA52013) from *Streptomyces longisporoflavus*, MtrB (GenBank accession no. CAK50798) from *Streptomyces argillaceus*, Atr30 (GenBank accession no. QBG38791) from *Streptomyces atratus* SCSIO ZH16, DrrB (GenBank accession no. AAA74718) from *Streptomyces peucetius*, Different color box represents different transmembrane region. The fluorescent blue box indicates the inside region, the fluorescent pink box indicates outside region and the fluorescent green indicates the transmembrane region. The two marked motifs were proposed functional similar to the conserved “EAA motif “of importers.

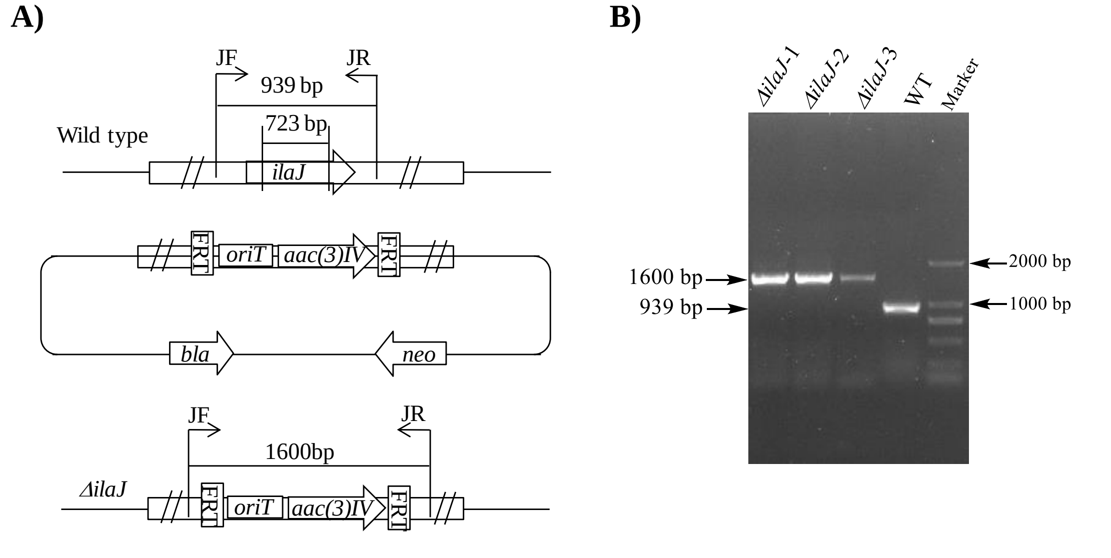

Figure S5. Disruption of *ilaJ* in wild type S. atratus SCSIO ZH16 via PCR-targeting. (A) Schematic representation for disruption of *ilaJ*. (B) PCR analyses of the WT strain and the *ilaJ* double-cross mutant carried out using the primers listed in Table S2. M: DNA molecular ladder; WT: using the genomic DNA of *S. atratus* SCSIO ZH16 as template; Δ*ilaJ*-1-3: using the genomic DNA of *ilaJ* mutant as template.

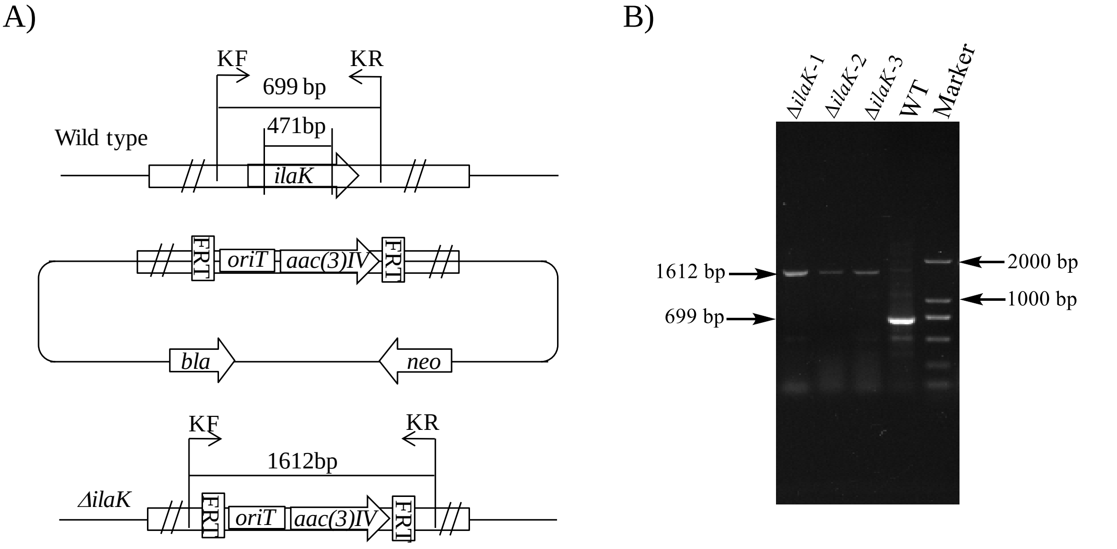

Figure S6. Disruption of *ilaK* in wild type S. atratus SCSIO ZH16 via PCR-targeting. (A) Schematic representation for disruption of *ilaK*. (B) PCR analyses of the WT strain and the *ilaK* double-cross mutant carried out using the primers listed in Table S2. M: DNA molecular ladder; WT: using the genomic DNA of *S. atratus* SCSIO ZH16 as template; Δ*ilaK*-1-3: using the genomic DNA of *ilaK* mutant as template.

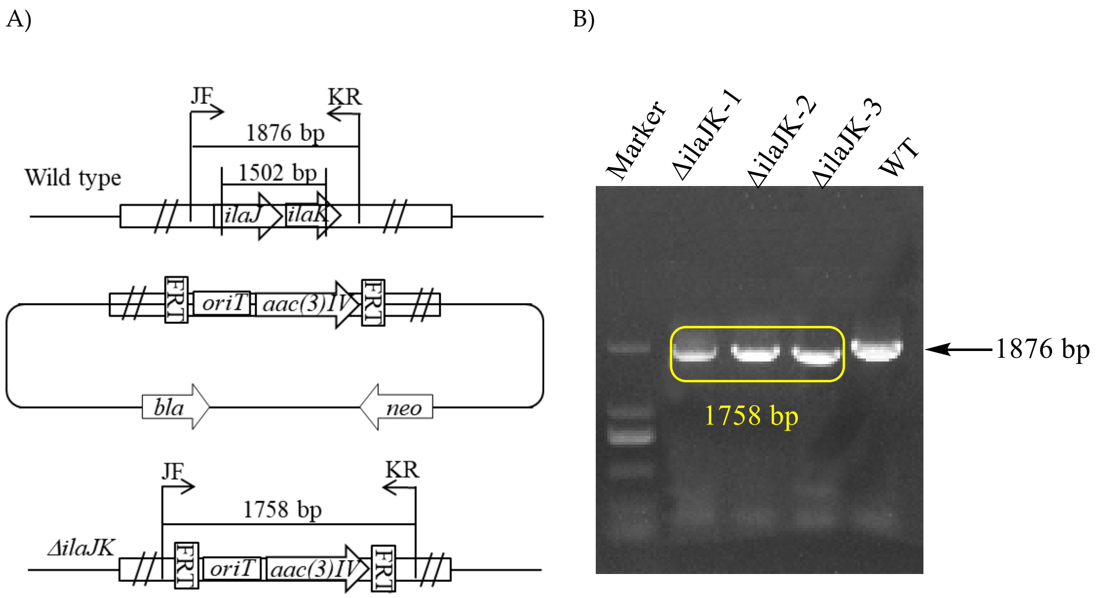

Figure S7. Disruption of *ilaJK* in wild type S. atratus SCSIO ZH16 via PCR-targeting. (A) Schematic representation for disruption of *ilaJK*. (B) PCR analyses of the WT strain and the *ilaJK* double-cross mutant carried out using the primers listed in Table S2. M: DNA molecular ladder; WT: using the genomic DNA of *S. atratus* SCSIO ZH16 as template; Δ*ilaJK*-1-3: using the genomic DNA of *ilaJK* mutant as template.

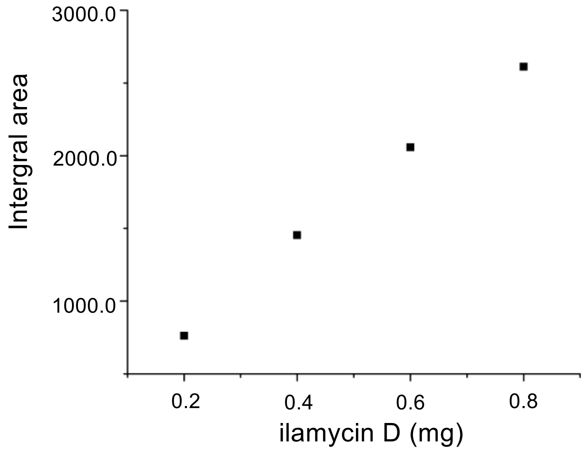

Figure S8. The standard curve of ilamycin D based on HPLC analysis.

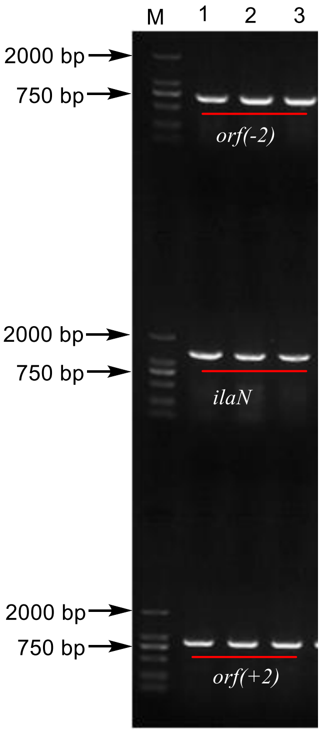

Figure S9. The PCR verification of the *ila* BGC heterologous expression conjugants with three pairs of primers listed in Table S2. M: DL2000 marker, 1-3: using the genomic DNA of the *ila* BGC successfully expressed conjugants as template.

References

1. Ma, J.; Huang, H.; Xie, Y.; Liu, Z.; Zhao, J.; Zhang, C.; Jia, Y.; Zhang, Y.; Zhang, H.; Zhang, T. Biosynthesis of ilamycins featuring unusual building blocks and engineered production of enhanced anti-tuberculosis agents. *Nat. Commun.* **2017**, *8*, 391.
2. Gomez-Escribano, J. P.; Bibb, M. J. Engineering Streptomyces *coelicolor* for heterologous expression of secondary metabolite gene clusters. *Microb. Biotechnol.* **2011**, *4*, 207–215.
3. Macneil, D. J.; Gewain, K. M.; Ruby, C. L.; Dezeny, G.; Gibbons, P. H.; Macneil, T., Analysis of *Streptomyces avermitilis* genes required for avermectin biosynthesis utilizing a novel integration vector. *Gene* **1992**, *111*, 61–68.
4. Datsenko, K. A.; Wanner, B. L., One-step inactivation of chromosomal genes in Escherichia coli K-12 using PCR products. *Proc. Nat. Acad. Sci. U SA* **2000**, *97*, 6640–6645.
5. Gust, B.; Chandra, G.; Jakimowicz, D.; Tian, Y. Q.; Bruton, C. J.; Chater, K. F., lambda red-mediated genetic manipulation of antibiotic-producing *Streptomyces*. *Adv. Appl. Microbiol.* **2004**, *54*, 107–128.
6. Paget, M. S. B.; Chamberlin, L.; Atrih, A.; Foster, S. J.; Buttner, M. J., Evidence that the extracytoplasmic function sigma factor sigma (E) is required for normal cell wall structure in *Streptomyces coelicolor* A3(2). *J. Bacteriol.* **1999**, *181*, 204–211.
7. Tu, J.; Li, S.; Chen, J.; Song, Y.; Fu, S.; Ju, J.; Li, Q., Characterization and heterologous expression of the neoabyssomicin/abyssomicin biosynthetic gene cluster from *Streptomyces koyangensis* SCSIO 5802. *Microbial Cell Factories* **2018**, *17*, 1–14.
